# GSA COVID-19 Task Force: Supporting Transitions to Online Teaching

**DOI:** 10.1093/geroni/igaa057.2945

**Published:** 2020-12-16

**Authors:** Joann Montepare, Judith Howe

**Affiliations:** 1 Lasell University, Newton, Massachusetts, United States; 2 Icahn School of Medicine at Mount Sinai, Bronx, New York, United States

## Abstract

GSA COVID-19 Task Force members collaborated with AGHE to develop multiple teaching briefs and biblio briefs on various topics to support faculty who had to adjust to online teaching quickly. Task Force members will review those documents, as well as the AGHE listening session to identify where educators and students need additional support.

